# Supplementary material for: Primary prophylaxis with mTOR inhibitor enhances T cell effector function and prevents heart transplant rejection during talimogene laherparepvec therapy of squamous cell carcinoma
Source: Nat Commun. 2024 Apr 30;15:3664. doi: 10.1038/s41467-024-47965-3 (PMC11063183; doi:10.1038/s41467-024-47965-3)
Supplement: Supplementary file 1 — Supplementary Information [file 41467_2024_47965_MOESM1_ESM.pdf]

Primary prophylaxis with mTOR inhibitor enhances T-cell effector function and prevents heart transplant rejection during talimogene laherparepvec therapy of squamous cell carcinoma

Victor Joo[1≠], Karim Abdelhamid[2≠], Alessandra Noto[1], Sofiya Latifyan[2], Federica Martina[1,] Douglas Daoudlarian[1], Rita Demicheli[2], Menno Pruijm[3], Solange Peters[2], Roger Hullin[4], Olivier Gaide[5], Giuseppe Pantaleo[1], Michel Obeid[1*]

[1]Centre Hospitalier Universitaire Vaudois (CHUV), University of Lausanne, Department of Medicine, Immunology and Allergy Division, Rue du Bugnon 46, CH-1011 Lausanne, Switzerland

[2]Centre Hospitalier Universitaire Vaudois (CHUV), University of Lausanne, Oncology Department, Rue du Bugnon 46, CH-1011 Lausanne, Switzerland

[3]Centre Hospitalier Universitaire Vaudois (CHUV), University of Lausanne, Department of Medicine, Nephrology Division, Rue du Bugnon 17, CH-1011 Lausanne, Switzerland

[4]Department of Heart-Vessels, Lausanne University Hospital CHUV, Lausanne, Switzerland

[5]Centre Hospitalier Universitaire Vaudois (CHUV), University of Lausanne, Dermatology Division, Rue du Bugnon 46, CH-1011 Lausanne, Switzerland

≠Equal contribution

***Corresponding author:**

Michel Obeid, MD, PhD

Centre Hospitalier Universitaire Vaudois (CHUV)

Immunology and Allergy Division, Rue du Bugnon 17, 1011 Lausanne, Switzerland

Email: michel.obeid@chuv.ch

Supplementary Figure 1

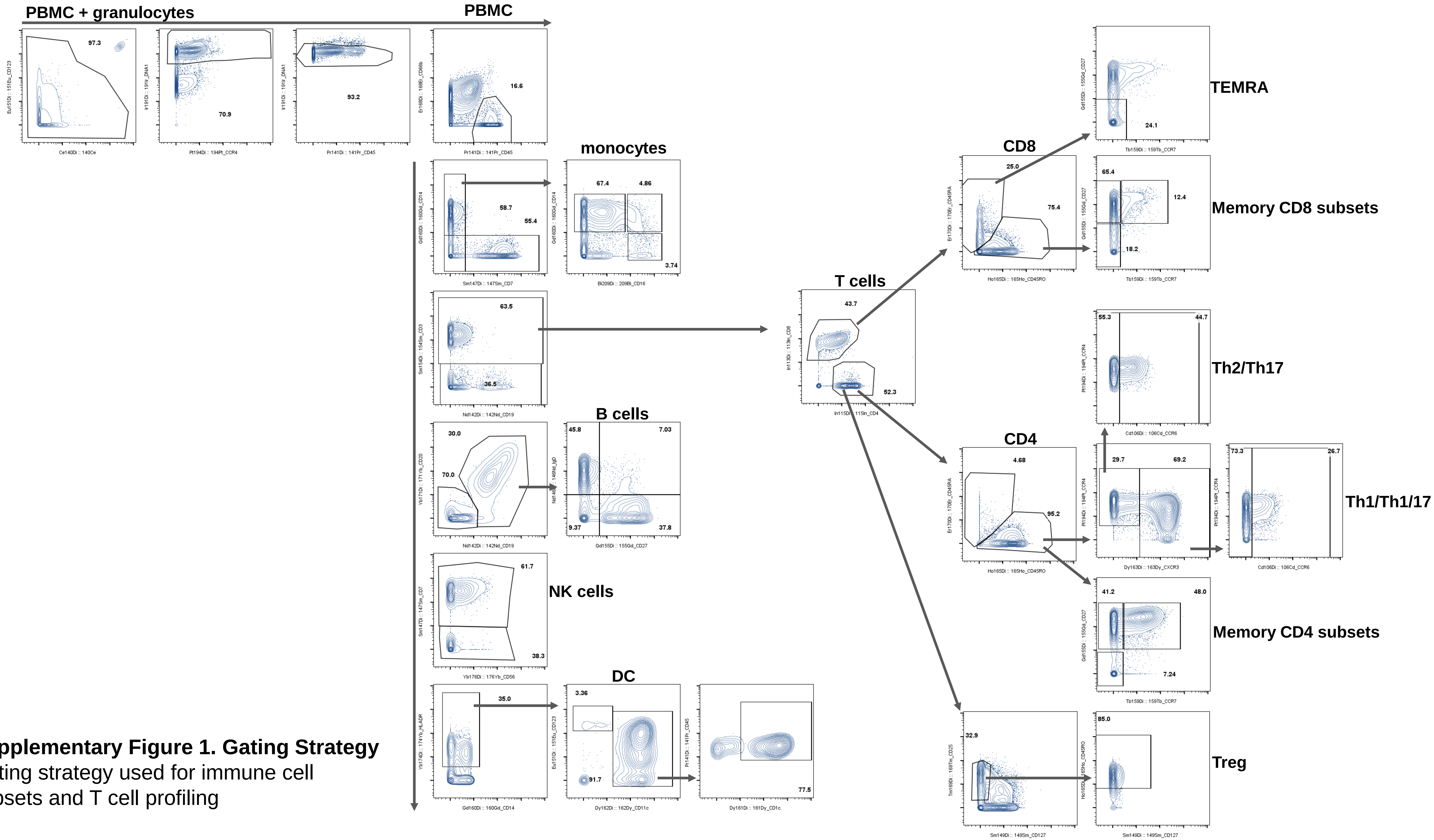

Supplementary Figure 1. Gating Strategy
Gating strategy used for immune cell subsets and T cell profiling

Supplementary Table 1. Antibodies used in this study

Immune Subsets Panel

Antibody-isotope	clone	distributor	dilution
CCR7-159	G043H7	Biolegend	1:200
CXCR5-164	J252D4	Biolegend	1:133.33
TCR γ/δ-156	B1	Biolegend	1:133.33
CD3-154	UCHT1	BD Biosciences	1:100
CD7-147	CD7-6B7	Biolegend	1:100
CXCR3-163	G025H7	Biolegend	1:83.33
CD45-141	HI30	Biolegend	1:83.33
CD141-111	1A4	BD Biosciences	1:500
CD69-112	FN50	Biolegend	1:500
CD16-209	3G8	Fluidigm/DVS	1:333.33
IgA2-116	A9604D2	SouthernBiotech	1:250
TCR α/β-173	T10b9.A-31	BD Biosciences	1:250
IgA1-148	B3506B4	SouthernBiotech	1:200
CCR4-194	L291H4	Biolegend	1:200
CD25-169	M-A251	Biolegend	1:200
CD4-115	RPA-T4	Biolegend	1:200
CD8-113	RPA-T8	Biolegend	1:200
CD1c-161	L161	Biolegend	1:200
CD21-152	BL13	Fluidigm/DVS	1:200
CD27-155	L128	Fluidigm/DVS	1:200
CD20-171	2H7	Fluidigm/DVS	1:200
PD1-175	EH12.2H7	Biolegend	1:200
CD45RO-165	UCHL1	Fluidigm/DVS	1:200
IgD-146	IA6-2	Fluidigm/DVS	1:200
CD62L-153	DREG-56	Fluidigm/DVS	1:200
IgM-172	MHM-88	Fluidigm/DVS	1:200
CD56-176	HCD56	Fluidigm/DVS	1:200
CD24-166	ML5	Fluidigm/DVS	1:181.18
ICOS-143	C398.4A	Biolegend	1:166.67
CD38-167	HIT2	Biolegend	1:166.67
CD127-149	A019D5	Fluidigm/DVS	1:166.67
CD31-145	WM59	Fluidigm/DVS	1:166.67
IgG3-144	HP6047	Biolegend	1:166.67
CD10-158	HI10a	Fluidigm/DVS	1:133.33
HLA-DR-174	L243	Fluidigm/DVS	1:133.33
CD66b-168	G10F5	BD Biosciences	1:133.33
CD45RA-170	HI100	Fluidigm/DVS	1:125
CD19-142	HIB19	Fluidigm/DVS	1:125
CD123-151	6H6	Fluidigm/DVS	1:125
CD11c-162	Bu15	Fluidigm/DVS	1:105.26
CD14-160	M5E2	Fluidigm/DVS	1:105.26
IgG1-150	G17-1	BD Biosciences	1:100
CCR6-106	11A9	BD Biosciences	1:100
IgG2-198	HP6002	Biolegend	1:66.67

T cell Phenotype Panel

Antibody-isotope	clone	distributor	dilution
CD3-154	UCHT1	BD Biosciences	1:100
CCR7-159	G043H7	Biolegend	1:100
CD7-147	CD7-6B7	Biolegend	1:100
CXCR5-164	J252D4	Biolegend	1:100
CXCR3-163	G025H7	Biolegend	1:100
CD45-89	HI30	Biolegend	1:100
CD4-115	RPA-T4	Biolegend	1:400
PD1-151	EH12.2H7	Biolegend	1:400
CD62L-153	DREG-56	Fluidigm/DVS	1:333.33
CD25-158	M-A251	Biolegend	1:333.33
CD16-209	3G8	Fluidigm/DVS	1:333.33
HLA-DR-174	L243	Fluidigm/DVS	1:333.33
CD38-167	HIT2	Biolegend	1:250
CCR4-110	L291H4	Biolegend	1:200
CD69-162D	FN50	Biolegend	1:200
CD45RO-165	UCHL1	Fluidigm/DVS	1:200
CCR6-141	11A9	BD Biosciences	1:200
CD27-155	L128	Fluidigm/DVS	1:200
CD8-113	RPA-T8	Biolegend	1:200
NKG2D-166	ON72	Fluidigm/DVS	1:166.67
CD45RA-170	HI100	Fluidigm/DVS	1:166.67
CD57-194	NK-1	Biolegend	1:142.86
CD19-142	HIB19	Fluidigm/DVS	1:142.86
TIGIT-144	MBSA43	Fluidigm/DVS	1:142.86
CD127-149	A019D5	Fluidigm/DVS	1:142.86
CD95-152	DX2	Thermo	1:142.86
CD56-176	HCD56	Fluidigm/DVS	1:142.86
ICOS-143	C398.4A	Biolegend	1:111.11
CD14-160	M5E2	Fluidigm/DVS	1:105.26
CD66b-168	G10F5	BD Biosciences	1:100
TIM-3-169	PCH101	Fluidigm/DVS	1:100
LAG3 -172	874501	R&D Systems	1:100
OX40-150	ACT35	Fluidigm/DVS	1:76.92
GRZb-171	GB11	Fluidigm/DVS	1:333.33
Bcl2-173	100	Biolegend	1:200
Perforin-175	B-D48	Fluidigm/DVS	1:166.67
Ki67-161	Ki67	Biolegend	1:142.86
